# Work addiction in Chinese white-collar workers: the psychometric properties of its measure and its comorbidity with general anxiety in network analysis

**DOI:** 10.1186/s40359-023-01247-7

**Published:** 2023-07-25

**Authors:** Ruimei SUN, Long W. LAM, Anise M. S. WU

**Affiliations:** 1 Department of Psychology, Faculty of Social Sciences, University of Macau, Avenida da Universidade, Taipa, Macao SAR China; 2 Center for Cognitive and Brain Sciences, University of Macau, Avenida da Universidade, Taipa, Macao SAR China; 3 Department of Management and Marketing, Faculty of Business Administration, University of Macau, Avenida da Universidade, Taipa, Macao SAR China

**Keywords:** Work addiction, Anxiety, Workaholism, Psychometric properties, Bergen Work Addiction Scale, Network analysis

## Abstract

**Background:**

Work addiction (WA) threatens occupation-related health in many countries including China. This research aims to evaluate the psychometric properties of the Chinese version of Bergen Work Addiction Scale (BWAS), the most common measure of WA, to facilitate relevant studies in Chinese workers. A network analysis was further conducted to identify central and bridge symptoms within the WA-anxiety network to improve intervention practices.

**Methods:**

A total of 694 Chinese white-collar workers completed an online questionnaire survey in March of 2022, and the responses to BWAS from a subsample of 50 participants one month after this survey were also collected.

**Results:**

The unidimensionality of BWAS was supported by results of exploratory factor analysis, exploratory graph analysis, and confirmatory factor analysis and we found satisfactory internal consistency and acceptable test-retest reliability. Multiple-group factor analyses confirmed the measurement invariance of BWAS across genders, districts (i.e., central China, eastern China, western China, and northeastern China), and age groups (i.e., young and middle-aged adults) while the convergent validity of BWAS was demonstrated by its significant correlations with Dutch Work Addiction Scale (*r =* 0.62, *p <* 0.001) and its criterion validity was indicated by its significant correlations with general anxiety, weekly work hours, and health status (*r =* -0.16 to 0.31, *p <* 0.001–0.01). Network analysis further revealed two central symptoms (WA-tolerance and WA-problems) and three bridge symptoms (WA-problems, WA-mood modification, and mouth dryness of general anxiety) maintaining the WA-anxiety comorbidity.

**Conclusions:**

Our findings suggest that BWAS is a valid measure of WA in Chinese workers and interventions should put special attention to the identified central and bridge symptoms underlying the WA-anxiety network.

## Background

### Work addiction

With its high prevalence of ranging from 8.3 to 23% [1], work addiction (WA) has caused substantial negative impacts on the functioning of not only employees [2] but also their organizations [3]. Although work is a necessary part of most people’s lives, WA may bring some benefits (e.g., organizational recognition and financial compensation) to the addicts and it is generally regarded as a type of behavioral addiction [4]. It shares commonalities (e.g., psychopathology correlates and addiction components) with other behavioral addictions [4, 5] and is characterized with an excessive focus on work, which is driven by uncontrollable motivation to work, as well as an abnormally high expense of energy on work, and thus compromising personal relationships, leisure activities, and/or health [5, 6]. Therefore, WA can be operationally defined as a persistent and maladaptive work pattern with major symptoms of addiction such as tolerance and withdrawal. Based on the components model of addiction [5, 7], WA should be defined with at least six symptoms, namely salience, mood modification, tolerance, withdrawal, conflict, and relapse. The components model of addiction, and its six symptoms, have been commonly used to define a specific behavioral addiction, particularly when its assessment tool was developed and/or evaluated, including Internet gaming disorder [8], social media addiction [9], and problematic series watching [10].

Guided by the components model of addiction, Andreassen et al. (2012) [6] developed a 7-item Bergen Work Addiction Scale (BWAS) to assess seven core features of WA (i.e., salience, mood modification, tolerance, withdrawal, conflict, relapse, and problems). BWAS has been translated from English into other languages including Hungarian [11], Polish [12], Danish [13], Italian [14], and Turkish [15]. It was also used in Chinese workers, who are highly vulnerable to both WA [16] and its negative consequences (e.g., psychological distress and impairment of physical health as well as social relationships) [17]. Unlike the translated scales of BWAS in other languages (e.g., Hungarian and Polish), the psychometric validation of the Chinese version of the scale has not been empirically conducted and thus its applicability to Chinese samples remained unknown. Moreover, general anxiety has also been commonly reported in workaholics [18, 19] but the relationship between general anxiety and WA has not yet been examined in Chinese workers. The present study hence has two aims: (i) conducting the first systematic psychometric investigation of BWAS in Chinese workers and (ii) utilizing network analysis to reveal the comorbidity of WA with general anxiety for enriching intervention strategies.

### The most commonly used measures of work addiction

Since Oates (1971) [20] initially introduced workaholism (i.e., compulsion or uncontrollable need to work incessantly), the pioneering research of WA has been contributed by four commonly used instruments, including Work Addiction Risk Test (WART) [21] including its revised form (i.e., Work Addiction Risk Test Revised [WART-R]) [22], Workaholism Battery (Work-BAT) [23], and Dutch Work Addiction Scale (DUWAS) [24]. However, scholars have also raised various concerns in terms of their theoretical bases and factor structures [1] [25]. The major criticism is that these measures were largely atheoretical and hence there are potential biases to certain symptoms of addiction. For examples, WART [26] measured only 5 features of WA based on the observation by clinicians who dealt with WA-related problems of clients and their families [21]. For the other two scales, only 3 workaholic’s features (i.e., high work involvement, motivation driven by inner pressure, and low work enjoyment) were assessed by Work-BAT [23] whereas 2 features were covered by DUWAS [24] based on compulsive tendencies of WART [26] and motivation driven by inner pressure of Work-BAT [23]. Therefore, the “components” of WA considered in these measures do not cover all core indicators of WA as an addictive behavior [5, 7]. Moreover, the factor structures of these measures were less stable in cross-cultural contexts. WART displayed different solutions of factors ranging from 3 among French workers [27] to 4 among Norwegian workers [28]. Work-BAT presented a 2-factor solution in Japanese workers [29] and Turkish working graduates [30] but a 5-factor solution in Chinese workers [31]. The 1-factor structure of DUWAS in Brazilian doctors [32] was also different from its original 2-factor structure in Dutch and Japanese workers [24]. Considering the aforementioned challenges for these popular measures, BWAS was developed within the addiction framework as proposed by Griffiths (2005, 2011) [5, 7].

### The Bergen work addiction scale

Andreassen et al. (2012) [6] developed a 7-item BWAS to measure WA by including all core elements of addiction based on the components model of addiction [5, 7]. The components model of addiction assumes that all addictions share common components (e.g., salience, tolerance, and withdrawal) and provides a multifactorial biopsychosocial approach to understand how the development of both substance use disorders and behavioral addictions can be influenced by psychological, physiological, social, and cultural factors that are unique to each individual. The unidimensionlity of BWAS has been supported across countries (e.g., Norway, Poland, Denmark, Italy, Turkey, and Hungary), with good reliability (e.g., internal consistency between 0.76 and 0.84 and test-retest reliability of 0.83) as well as convergent, discriminant, criterion, and predictive validity (i.e., correlated with WART, Work-BAT, DUWAS, weekly work hours, work engagement, neuroticism, attention deficit hyperactivity disorder, psychological distress, low life quality, and future work-family conflict respectively) [6, 11–15].

While BWAS has been used among Chinese employees of different industries with preliminary evidence for 1-factor structure [33] and good internal consistency (α = 0.80–0.89) [33–35], its applicability to the Chinese working population has not been systematically and psychometrically analyzed. To address this knowledge gap, the first aim of this study is to examine not only the construct validity (i.e., unidimensionality) of BWAS in cross-occupational white-collar workers in China but also its internal consistency, test-retest reliability, as well as convergent and criterion validity. The convergent validity of BWAS will be examined via its correlation with DUWAS score because both scales were developed for WA and prior studies have shown moderate to high correlations between them (*r* = 0.38 to 0.61, *p* < 0.001) among workers in Italy [14] and Turkey [15]. Since long work hours, high anxiety level, and poor health are commonly observed among workaholics [12, 13, 15, 36], we will also analyze if BWAS is positively correlated with general anxiety and weekly work hours, and negatively correlated with self-rated health status in order to understand its criterion validity. In addition to testing the factor-level properties, the second aim of this study is applying network analysis to identify both the central and bridge symptoms connecting WA and general anxiety [37].

### Network analysis between work addiction and general anxiety

People with general anxiety experience a prolonged and unpleasant emotional state, involving the feelings of worry and fear that arise from perceiving or evaluating a wide range of situations or events as potential threats or dangers [38, 39]. An uncontrollable and excessive level of general anxiety is often referred as generalized anxiety disorder, a common mental illness in modern societies. WA and general anxiety have been consistently reported to be moderately and positively correlated [15, 40, 41] but the details underlying their correlation remains underexplored. Which individual symptoms are working in the WA-anxiety relation have not been ever investigated in the existing literature. Network analysis is a statistical approach allowing visual depiction of the complex associations among individual symptoms underlying a latent factor of a psychological disorder or condition using the disease model [37]. Specifically, it can be used to identify both central symptom(s) (quantified by its partial correlations with other symptoms within the whole network) and bridge symptom(s) (quantified by pathways it connects to symptoms of another condition) maintaining the “comorbidity” of different conditions [37]. The present study aims to apply this statistical technique to reveal how the connection between WA and general anxiety is maintained by the central and bridge symptoms in the network. This exploratory investigation broadens our knowledge on not only the complex interactions between individual symptoms underlying the co-occurring WA-anxiety mechanism but also cost-effective workplace intervention strategies to mitigate WA and general anxiety simultaneously by focusing on these comorbid symptoms among working adults.

### The current study

Using a sample of white-collar workers from various industries in China, this study was the first to evaluate multiple psychometric properties (i.e., construct validity, reliabilities, and convergent and criterion validity) of BWAS, which is a short and useful tool for WA, in the Chinese context. Findings of our study would help provide first-hand evidence for or against the use of such popular measurement tool in the Chinese workers. Moreover, this study was also the first to identify the comorbidity of WA with general anxiety by network analysis. The results of this network analysis may contribute to better insights for interventions to buffer both WA and general anxiety simultaneously and in turn improve their cost-effectiveness by targeting on these central and bridge symptoms connecting WA and general anxiety.

## Methods

### Participants and procedures

After obtaining the ethics approval from the ethics committee of the Department of Psychology at the corresponding author’s affiliated university, we conducted a web-based questionnaire survey through Credamo, a professional data platform which has more than 2.8 million registered adult participants from different provinces and occupations and proper quality assurance procedures. For this study, Credamo distributed the questionnaire to the eligible participants (i.e., Chinese white-collar workers who are 18 or above and have one year or more work experience) in March of 2022. Before starting to fill in the questionnaire, participants were informed about the study aim and their rights. Participants were also requested to provide their consent to participate in the study voluntarily and anonymously. Then, among participants who completed the survey, Credamo only selected those who successfully passed the attention check item (i.e., “Please select ‘1 = totally disagree’ as the answer of the item “I feel very relaxed during the break”). Finally, we received the dataset of a valid sample from Credamo for our data analysis, which was composed of 694 Chinese adult white-collar workers with one year or more work experience who passed the attention check item.

This valid sample consisted of 694 Chinese white-collar workers (58.8% females; *M*_*age*_ = 32.07, *SD* = 5.61, *range* from 24 to 55 years) with no missing values for data analysis of our study. The demographic characteristics (i.e., gender, age, job tenure, weekly work hours, and industry) of our worker sample are displayed in Table 1. In this sample, more than half of workers (55.7%) aged between 30 and 39 years and was in their current job for 5–10 years. About one-fourth (26.1%) reported job tenure of less than 5 years, with the remaining (23.3%) reported job tenure of more than10 years. More than 28.9% reported working longer than 50 h weekly. Around one-third of workers came from the manufacturing industry (28.8%), followed by culture/sport/education industry (13.8%), service industry (12.7%), information technology industry (12.2%), banking/finance (11.7%), and others (including construction, medical insurance, transportation, and government or non-profit organization).

**Table 1:** Demographic characteristics of the overall participants (*N* = 694)

Variables	*Categories*	*N*	*%*
1. Gender	Male	286	41.2
	Female	408	58.8
2. Age (*years*)^#^	24 or below	12	1.7
	25–29	232	33.4
	30–34	284	40.9
	35–39	103	14.8
	40–49	48	6.9
	50 or above	15	2.2
3. Job tenure	Less than 1 year	0	0
	1–3 years	78	11.2
	3–5 years	103	14.8
	5–10 years	351	50.6
	More than 10 years	162	23.3
4. Weekly work hours^#^	39 or below	32	4.6
	40–49	462	66.6
	50–59	133	19.2
	60–69	49	7.1
	70 or above	18	2.6
5. Industry	Manufacturing industry	200	28.8
	Culture/Sport/Education industry	96	13.8
	Service industry	88	12.7
	Information technology industry	85	12.2
	Banking/Finance industry	81	11.7
	Construction industry	43	6.2
	Medical insurance industry	21	3.0
	Transportation industry	19	2.7
	Others (e.g., Government or non-profit organizations)	61	8.8

*Note.* ^#^We collected participants’ continuous responses on age and weekly work hours and then recoded them as categorical variables in Table 1.

Guided by the Bonett’s (2002) [42] sample size formula, which consists of number of items of the scale, expected Cronbach’s α, desired precision, and confidence level to estimate the required sample size, a web-based sample size calculator (https://wnarifin.github.io/ssc/ssalpha.html; [43]) was developed and was used in this study to determine the minimum sample size required for achieving a Cronbach’s α = 0.8 with a precision of 0.1 and a 95% confidence interval of [0.70, 0.90] for 7-item BWAS. Result of this calculator suggests that at least 42 participants are required for the desired internal consistency (i.e., Cronbach’s α) for BWAS. As such, our sample size (N = 694), even after split-half procedure, met the requirement. In addition to internal consistency test, we also explored the test-retest reliability of BWAS with a smaller follow-up sample (n = 50; one month after the online survey, i.e., April 2022) using Pearson correlation. Based on the advice from DeVet et al. (2011) [44], a sample size of 50 is a minimum one required for examining the test-retest reliability of a scale.

### Measures

#### The Bergen Work Addiction Scale

The original English version of BWAS [6] was translated into Chinese by the first author of the present study and the Chinese version was then translated back into English by another two bilingual psychologists. By comparing both the original and back-translated English versions, the translated Chinese items were finalized and unanimously agreed by the three translators. A 5-point Likert scale (1 = *never*, 5 = *always*) was provided for participants to respond to each BWAS item. A sample item is “How often during the last year have you spent much more time working than initially intended?”. Higher mean scores represented higher levels of WA.

#### The Dutch Work Addiction Scale

The 10-item DUWAS [24], which has been used in Chinese adult samples [16, 45], comprises 5 items for working excessively (e.g., “I find myself continuing to work after my coworkers have called it quits.”) and 5 items for working compulsively (e.g., “I feel that there is something inside me that drives me to work hard.”). Respondents were asked to rate this scale on a 4-point Likert scale ranging from 1 (*totally disagree*) to 4 (*totally agree*). Higher mean scores represented higher levels of WA. The Cronbach’s α of DUWAS in the present study was 0.81.

#### General anxiety

General anxiety was assessed by the 7-item anxiety subscale (e.g., “I was worried about situations in which I might panic and make a fool of myself.”) from the Chinese version [46] of the Depression Anxiety Stress Scales (DASS21) [47]. All items were rated on a 4-point Likert scale from 0 (*did not apply to me at all*) to 3 (*applied to me very much or most of the time*). Higher mean scores indicated higher levels of general anxiety. The Cronbach’s α of the subscale was 0.78 in the present study.

#### Self-rated health status

The self-rated health status was assessed by single question “In general, how would you rate your current health status?” which is recommended by the World Health Organization (1996) [48] and the European Network for the Calculation of Health Expectancies [49]. The answers include: 1 = *very good*, 2 = *good*, 3 = *fair*, 4 = *bad*, and 5 = *very bad*. After reverse coding, a higher score represented better health status.

### Statistical analyses

#### Examination of the psychometric properties of the Chinese version of BWAS

All statistical analyses involved in this study were conducted in R (version 4.2.1). After randomly dividing all participants into sample A (*N* = 347) and sample B (*N* = 347), sample A was used for parallel analysis, exploratory factor analysis (EFA) using the promax rotation, and exploratory graph analysis (EGA) [50, 51] which used the *graphical least absolute shrinkage and selection operator* (glasso; [52]) in the ‘EGAnet’ package [53] to assess the dimensionality of psychological instrument. Sample B was used for confirmatory factor analysis (CFA; for testing the construct validity of BWAS), internal consistency (i.e., Cronbach’s α) test, Pearson correlation analyses (for evaluating test-retest reliability as well as convergent and criterion validity), and multi-group CFA (for testing the measurement invariance).

In both EFA and CFA, we adopted Maximum Likelihood (ML) estimation because the normal distribution assumption of the 7 items of BWAS was met by the absolute values of (i) item skewness (range between 0.152 and 0.825 in sample A and 0.073–0.924 in sample B) < 2 and (ii) item kurtosis (range between 0.088 and 0.796 in sample A and 0.150–0.778 in sample B) < 7 [54]. To examine whether a model shows an acceptable fit to the data, we followed Hu and Bentler’s guideline (1999) [55]: comparative fit index (CFI > 0.90), Tucker Lewis Index (TLI > 0.90), root mean square error of approximation (RMSEA < 0.08), and standardized root mean square residual (SRMR < 0.08).

In sample B, a series of multi-group CFA using ML estimation were performed to examine the four levels of measurement invariance (i.e., M1: configural invariance; M2; metric invariance; M3: scalar invariance; M4: strict invariance) of BWAS in three background variables: two genders (i.e., 153 males and 194 females), four major economic districts (i.e., 88 participants from central China, 200 from eastern China, 36 from western China, and 23 from northeastern China) [56], and two age groups (i.e., 264 young adults aged between 18 and 34 years and 83 middle-aged adults between 35 and 59 years [57, 58]). By comparing the goodness of fit (using CFI and RMSEA) of these four nested measure models (from M1 to M4), |ΔCFI| < 0.01 and |ΔRMSEA| < 0.015 [59] would support these measurement invariances.

#### Network analysis

Among all participants (N = 694), the specific packages in R (version 4.2.1) were used to conduct network analysis, in which node represents the symptom and edge represents the partial correlation between two nodes while controlling for other nodes [60, 61]. The network model was estimated in the ‘qgraph’ package [62] by the *Extended Bayesian Information Criterion glasso* (EBICglasso) which could improve the interpretability and predictive accuracy of the network model [52].

To identify central symptoms, the expected influence (1-step), defined as the sum of edges between a node and all nodes within the network [61], was visualized by ‘centralityPlot’ and ‘centralityTable’ functions in the ‘qgraph’ package [62]. A higher EI (1-step) value indicates a more influential central node within the network. To identify bridge symptoms, the bridge expected influence (1-step), defined as the sum values of edges between a node in one psychological condition and all nodes in another condition [63], was estimated by the ‘bridge’ function in ‘networktools’ package [64]. A higher value of bridge EI (1-step) indicates a more influential bridge node in the connection between WA and general anxiety.

The ‘bootnet’ package [65] was further applied to estimate the centrality stability and edge-weights accuracy. The central stability (CS) coefficient was calculated by the ‘caseDroppingBoot’ function with 1000 iterations. Prior study has suggested the CS coefficient above 0.25 as acceptable and above 0.5 to be preferable [66]. The accuracy of edge-weights at 95% confidence intervals (CIs) was estimated by the ‘nonParametricBoot’ function with 1000 iterations. Larger CIs mean less accurate edge estimation while narrower CIs mean more accurate estimation of the edges [67].

## Results

### Common method bias test

We conducted Harman’s single-factor test to investigate the common method bias among 25 items of four key variables (i.e., BWAS, DUWAS, general anxiety, and self-rated health status). The results indicated that there were 5 factors with the original root more than 1 and the cumulative variance explained by the first factor was 25.39%, which is less than the critical value (40%) of reaching severe common method bias [68].

### Psychometrics properties of the Chinese version of BWAS

#### Exploratory factor structure

**Fig. 1:**
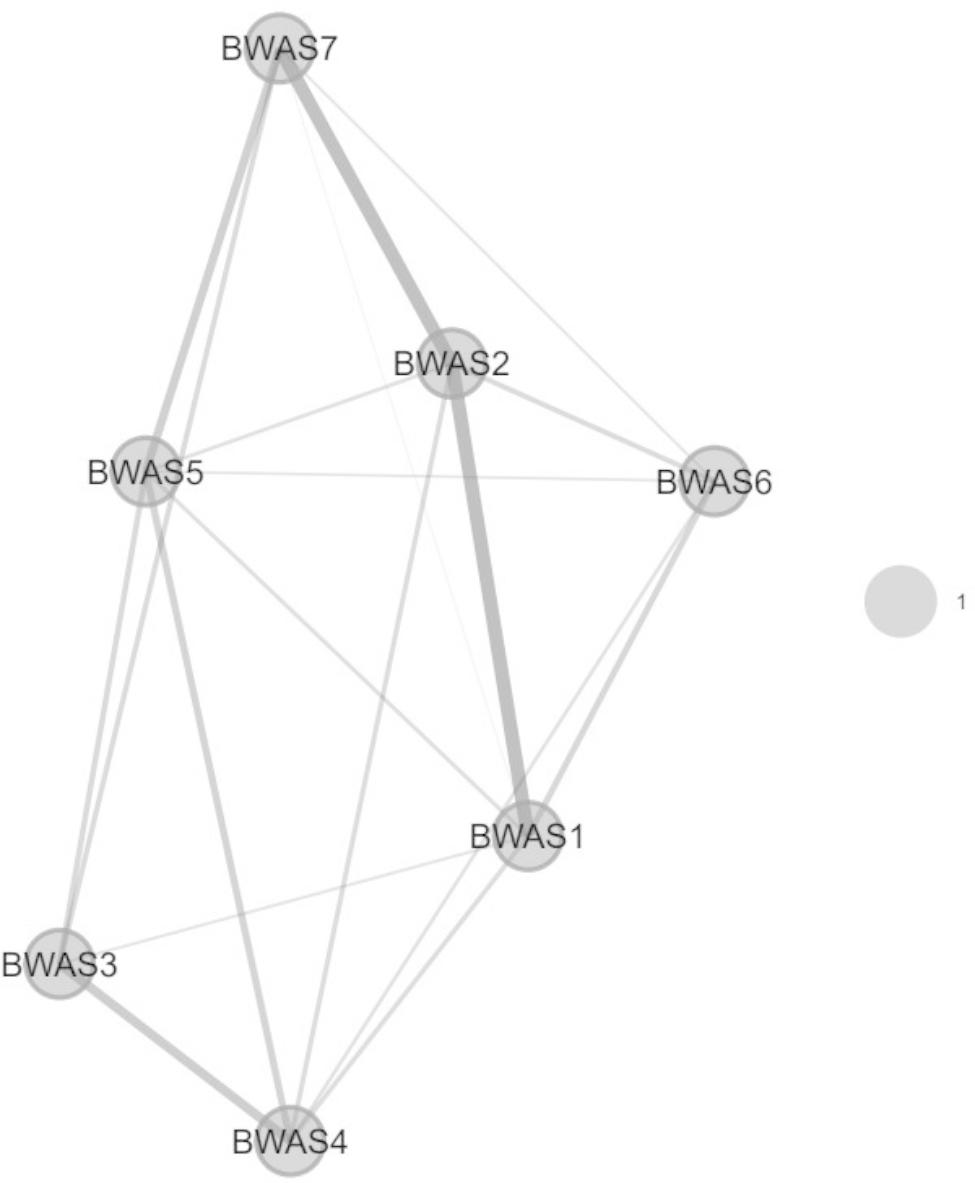
The one-factor structure of BWAS identified by exploratory graph analysis. Note. BWAS = Bergen Work Addiction Scale

Both the results of both EFA and EGA supported the unidimensionality of BWAS. The EFA model identifies a one-factor structure with eigenvalues > 1 (3.059), which is consistent with the result of parallel analysis which also suggests extracting one factor from BWAS. As shown in Table 2, the standardized factor loadings of each item ranged between 0.452 and 0.722, which are above 0.40 and considered as acceptable [69]. Moreover, EGA also finds all items of BWAS belonging to one cluster, indicating a one-factor structure of BWAS (Fig. 1).

**Table 2:** EFA and CFA of BWAS based on Sample A (*N* = 347) and Sample B (*N* = 347) respectively

Items *How often in the last year have you…*	Addiction components	Factor Loadings in EFA	Factor Loadings in CFA
1. Thought of how you could free up more time to work?	Salience	0.640	0.733^***^
2. Spent much more time working than initially intended?	Tolerance	0.722	0.764^***^
3. Worked in order to reduce feelings of guilt, anxiety, helplessness and depression?	Mood modification	0.452	0.579^***^
4. Been told by others to cut down on work without listening to them?	Relapse	0.570	0.704^***^
5. Become stressed if you have been prohibited from working?	Withdrawal	0.611	0.602^***^
6. Deprioritized hobbies, leisure activities, and exercise because of your work?	Conflict	0.460	0.571^***^
7. Worked so much that it has negatively influenced your health?	Problems	0.622	0.586^***^

*Note*. BWAS = Bergen Work Addiction Scale, Exploratory factor analysis = EFA, Confirmatory factor analysis = CFA. All items were rated on a 5-point Likert scale (1 = *Never*, 2 = *Rarely*, 3 = *Sometimes*, 4 = *Often*, 5 = *Always*)

#### Construct validity

Results of CFA show that the one-factor model of BWAS fits well with our data: *χ*^*2*^(14) = 33.162, *χ*^*2*^*/df* = 2.37, *p* < 0.01, *CFI* = 0.974, *TLI* = 0.961, *SRMR* = 0.034, and *RMSEA* = 0.063 with *90% CI* = [0.035, 0.091]. As shown in Table 2, the standardized factor loadings of each item ranged from 0.571 to 0.764. Therefore, the construct validity of BWAS was supported in this sample.

#### Measurement invariance across genders, districts, and age groups

Table 3 indicated a good model fit of the Chinese version of BWAS on the configural, metric, scalar, and strict levels across genders (*CFI* = 0.971 to 0.977, *TLI* = 0.965 to 0.974, *RMSEA* = 0.051 to 0.059, *SRMR* = 0.035 to 0.059). By comparing the model fits among these four levels, the measurement invariance across genders was supported at the strict level (M2-M1: |ΔCFI| = 0.005, |ΔRMSEA| = 0.000; M3-M2: |ΔCFI| = 0.001, |ΔRMSEA| = 0.006; M4-M3: |ΔCFI| = 0.002, |ΔRMSEA| = 0.002), as suggested by the cutoff of |ΔCFI| < 0.01 and |ΔRMSEA| < 0.015 [59]. Similar results in Tables 4 and 5 also supported the measurement invariance of BWAS across four major economic districts in China and two age groups (i.e., young adults and middle-aged adults) at the strict level respectively.

**Table 3:** Measurement invariance of BWAS across genders based on Sample B (*N* = 347)

Model		*χ* ^*2*^	*df*	*p*	CFI	TLI	RMSEA	SRMR	△*χ*^*2*^	△*df*	△*p*	|△CFI|	|△RMSEA|
M1: Configural invariance	44.948		28	0.022	0.977	0.966	0.059	0.035	-	-	-	-	-
M2: Metric invariance	54.697		34	0.014	0.972	0.965	0.059	0.055	9.749	6	0.136	0.005	0.000
M3: Scalar invariance	59.863		40	0.022	0.973	0.972	0.053	0.058	5.166	6	0.523	0.001	0.006
M4: Strict invariance	68.520		47	0.022	0.971	0.974	0.051	0.059	8.658	7	0.278	0.002	0.002

*Note*. BWAS = Bergen Work Addiction Scale; CFI = comparative fit index; TLI = Tucker-Lewis index; RMSEA = root mean square error of approximation; SRMR = standardized root mean square residual. Sample B includes 153 males and 194 females

**Table 4:** Measurement invariance of BWAS across four major economic districts in China based on Sample B (*N* = 347)

Model		*χ* ^*2*^	*df*	*p*	CFI	TLI	RMSEA	SRMR	△*χ*^*2*^	△*df*	△*p*	|△CFI|	|△RMSEA|
M1: Configural invariance	85.264		56	0.007	0.961	0.942	0.078	0.044	-	-	-	-	-
M2: Metric invariance	101.722		74	0.018	0.963	0.958	0.066	0.064	16.459	18	0.561	0.002	0.012
M3: Scalar invariance	127.044		92	0.009	0.954	0.958	0.066	0.071	25.321	18	0.116	0.009	0.000
M4: Strict invariance	142.754		113	0.031	0.961	0.971	0.055	0.077	15.710	21	0.786	0.007	0.011

*Note*. BWAS = Bergen Work Addiction Scale; CFI = comparative fit index; TLI = Tucker-Lewis index; RMSEA = root mean square error of approximation; SRMR = standardized root mean square residual. Sample B includes 88 participants from central China, 200 from eastern China, 36 from western China, and 23 from northeastern China

**Table 5:** Measurement invariance of BWAS among young adults and middle-aged adults based on Sample B (*N* = 347)

Model		*χ* ^*2*^	*df*	*p*	CFI	TLI	RMSEA	SRMR	△*χ*^*2*^	△*df*	△*p*	|△CFI|	|△RMSEA|
M1: Configural invariance	57.813		28	0.001	0.960	0.940	0.078	0.040	-	-	-	-	-
M2: Metric invariance	60.502		34	0.003	0.965	0.956	0.067	0.045	2.689	6	0.847	0.004	0.011
M3: Scalar invariance	67.200		40	0.005	0.964	0.962	0.063	0.047	6.698	6	0.350	0.001	0.004
M4: Strict invariance	70.875		47	0.014	0.968	0.972	0.054	0.049	3.675	7	0.816	0.004	0.008

*Note*. BWAS = Bergen Work Addiction Scale; CFI = comparative fit index; TLI = Tucker-Lewis index; RMSEA = root mean square error of approximation; SRMR = standardized root mean square residual. Sample B includes 264 young adults aged between 18–34 years and 83 middle-aged adults between 35–59 years

#### Reliabilities

All items of BWAS were significantly and positively correlated with each other (*r* = 0.28 to 0.59, *p* < 0.001), with a good internal consistency (Cronbach’s α = 0.83) among sample B (n = 347). Moreover, BWAS’s one-month test-retest reliability was 0.71 among the 50 follow-up participants (58.0% females; *M*_*age*_ = 31.54, *SD* = 4.52, *range* from 25 to 47 years). Among this follow-up sample (n = 50) one month after the online survey, the internal consistency of BWAS was also good (Cronbach’s α = 0.84).

#### Convergent and criterion validity

The descriptive statistics and inter-correlations among all variables are displayed in Table 6. The score of BWAS was highly and positively correlated with that of DUWAS (*r* = 0.62, *p* < 0.001), which supported the convergent validity of BWAS. As expected, general anxiety and work hours were significantly and positively correlated with BWAS while BWAS is significantly and negatively correlated with self-rated health status (*r* = -0.16 to 0.31, *p* < 0.001–0.01), which supported the criterion validity of BWAS.

**Table 6:** Correlation among all variables based on Sample B (N = 347)

	*M*	*SD*	1	2	3	4	5	6	7	8
1. BWAS	3.09	0.78	-							
2. DUWAS	2.74	0.53	0.62^***^	-						
3. General anxiety	0.55	0.48	0.31^***^	0.24^***^	-					
4. Self-rated health status	4.03	0.70	− 0.16^**^	0.01	− 0.31^***^	-				
5. Gender^#^	1.56	0.50	0.12^*^	0.07	0.06	− 0.05	-			
6. Age	31.96	5.27	0.09	0.10	− 0.10	− 0.08	− 0.07	-		
7. Job tenure^#^	3.85	0.93	0.05	0.12^*^	− 0.13^*^	0.007	− 0.02	0.75^***^	-	
8. Weekly work hours	46.88	7.98	0.25^***^	0.20^***^	0.19^***^	− 0.16^**^	− 0.09	− 0.07	-0.10	-

*Note*. BWAS = Bergen Work Addiction Scale, DUWAS = Dutch Work Addiction Scale; M = Mean, SD = Standard deviation; ^#^Categorical variables: (Gender: 1 = *Male*, 2 = *Female*; Job tenure: 1 = *Less than 1 year*; 2 = *1–3 years*, 3 = *3–5 years*; 4 = *5–10 years*; 5 = *More than 10 years*); ^*^p < 0.05, ^**^p < 0.01. ^***^p < 0.001

### Network analysis

#### Central symptoms and bridge symptoms

**Fig. 2:**
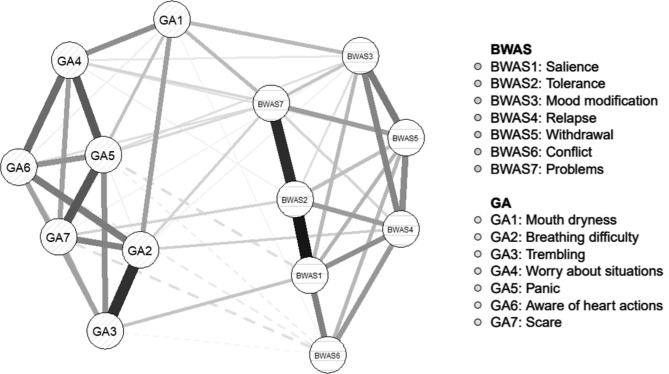
Network model of BWAS-GA symptoms. Note. BWAS = Bergen Work Addiction Scale, GA = General anxiety. Solid edges represent positive correlations, and dashed edges represent negative correlations. The thickness of edges indicates the strength of correlations between symptoms and a thicker edge indicated a stronger correlation between two symptoms

The network model of the symptoms of both WA and general anxiety (GA) was shown in Fig. 2.

**Fig. 3:**
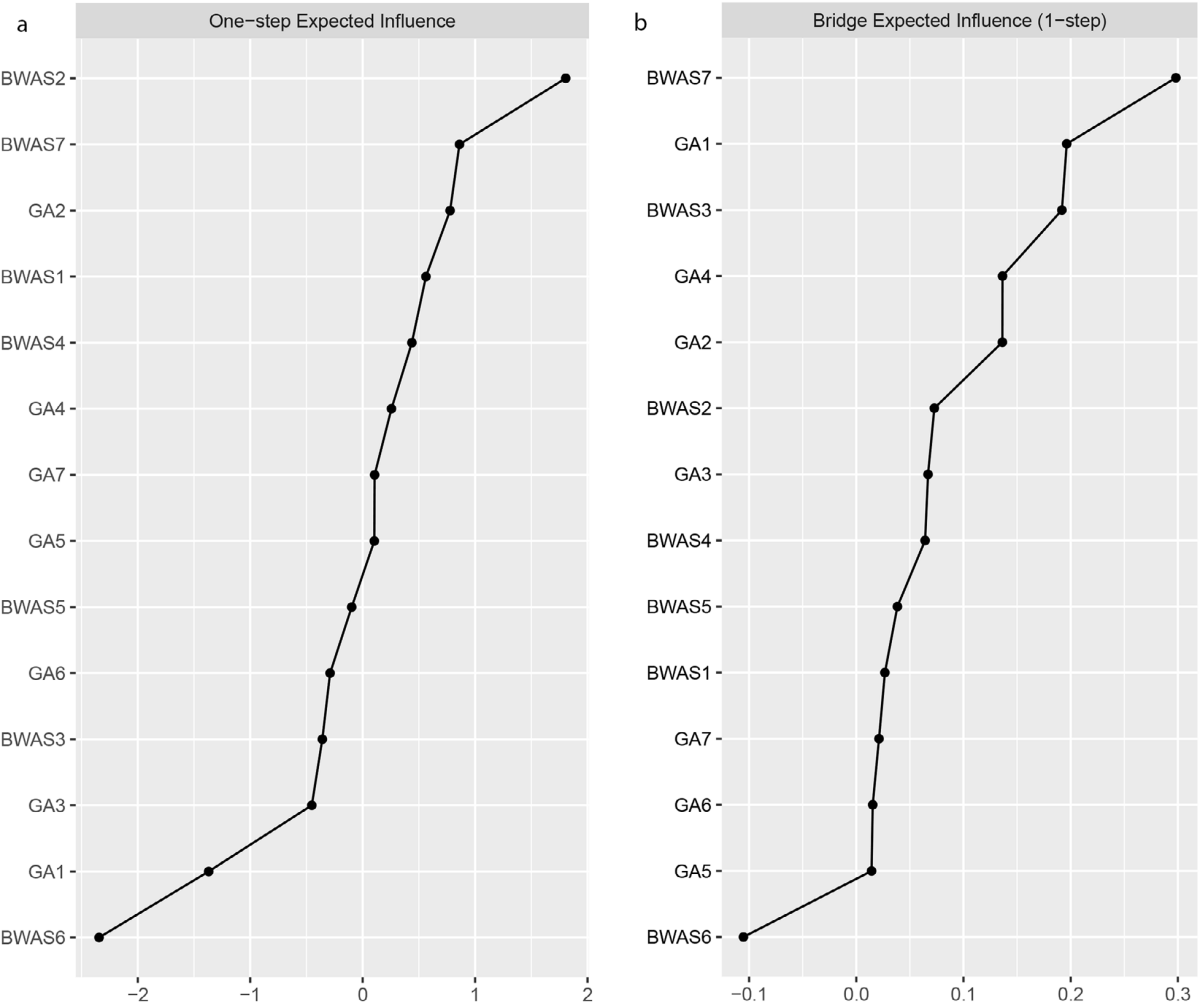
**a**. Expected Influence (1-step) of all symptoms. **b**. Bridge Expected Influence (1-step) of all symptoms

Among all 14 symptoms in the network, the EI (1-step) indices reported in both Fig. 3a; Table 7 indicated that WA-tolerance (BWAS2: spent much more time working than initially intended) was the most central symptom, followed by WA-problems (BWAS7: worked so much that it has negatively influenced your health).

**Table 7:** The EI (1-step) indices and bridge EI (1-step) indices of all symptoms within the network

Nodes	Items	EI (1-step)	Bridge EI (1-step)
BWAS1	Thought of how you could free up more time to work	0.563	0.027
BWAS2	Spent much more time working than initially intended	1.806	0.073
BWAS3	Worked in order to reduce feelings of guilt, anxiety, helplessness and depression	-0.358	0.192
BWAS4	Been told by others to cut down on work without listening to them	0.438	0.064
BWAS5	Become stressed if you have been prohibited from working	-0.097	0.038
BWAS6	Deprioritized hobbies, leisure activities, and exercise because of your work	-2.344	-0.105
BWAS7	Worked so much that it has negatively influenced your health	0.861	0.298
GA1	I was aware of dryness of my mouth	-1.369	0.196
GA2	I experienced breathing difficulty (e.g., excessively rapid breathing, breathlessness in the absence of physical exertion)	0.777	0.136
GA3	I experienced trembling (e.g., in the hands)	-0.452	0.067
GA4	I was worried about situations in which I might panic and make a fool of myself	0.256	0.136
GA5	I felt I was close to panic	0.104	0.014
GA6	I was aware of the action of my heart in the absence of physical exertion (e.g., sense of heart rate increase, heart missing a beat)	-0.290	0.015
GA7	I felt scared without any good reason	0.106	0.021

*Note*. Expected influence (1-step) = EI (1-step). BWAS = Bergen Work Addiction Scale, GA = General anxiety

According to the bridge EI (1-step) indices shown in Fig. 3b, WA-problems and WA-mood modification (BWAS3: worked in order to reduce feelings of guilt, anxiety, helplessness and depression) and GA-mouth dryness (DASS-GA1: I was aware of dryness of my mouth) were identified as the strongest bridge symptoms which connecting WA and general anxiety. Particularly, as shown in Table 8, the partial correlation between WA-problems and GA-mouth dryness and that between WA-mood modification and GA-mouth dryness were the strongest (*r* = 0.09).

**Table 8:** Correlation matrices: edge weights of all symptoms

	BWAS1	BWAS2	BWAS3	BWAS4	BWAS5	BWAS6	BWAS7	GA1	GA2	GA3	GA4	GA5	GA6	GA7
BWAS1	-													
BWAS2	0.32	-												
BWAS3	0.09	0.04	-											
BWAS4	0.15	0.13	0.17	-										
BWAS5	0.10	0.08	0.18	0.16	-									
BWAS6	0.17	0.11	0.00	0.14	0.10	-								
BWAS7	0.02	0.29	0.08	0.05	0.13	0.06	-							
GA1	0.00	0.00	0.09	0.00	0.00	0.01	0.09	-						
GA2	0.00	0.00	0.01	0.06	0.00	0.00	0.06	0.13	-					
GA3	0.08	0.00	0.00	0.00	0.00	-0.01	0.00	0.00	0.29	-				
GA4	0.00	0.01	0.03	0.00	0.04	0.00	0.05	0.15	0.00	0.00	-			
GA5	-0.05	0.00	0.04	0.00	0.00	-0.03	0.05	0.03	0.02	0.15	0.22	-		
GA6	0.00	0.00	0.00	0.00	0.00	-0.03	0.05	0.02	0.18	0.12	0.20	0.15	-	
GA7	0.00	0.06	0.01	0.00	0.00	-0.05	0.00	0.08	0.17	0.11	0.13	0.23	0.08	-

*Note*. BWAS = Bergen Work Addiction Scale, GA = General anxiety

#### Network stability and accuracy

**Fig. 4:**
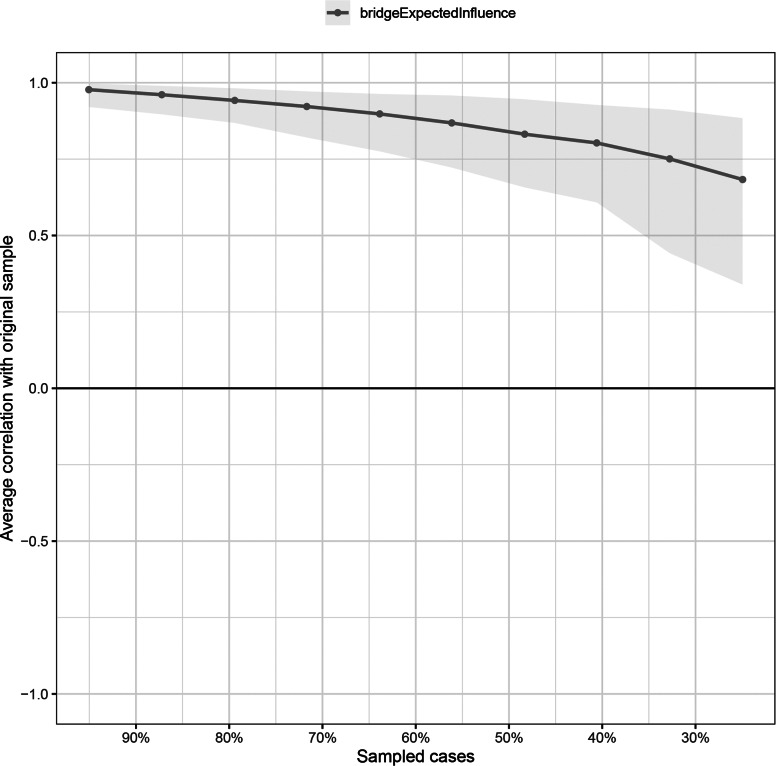
Stability of bridge expected influence indices

**Fig. 5:**
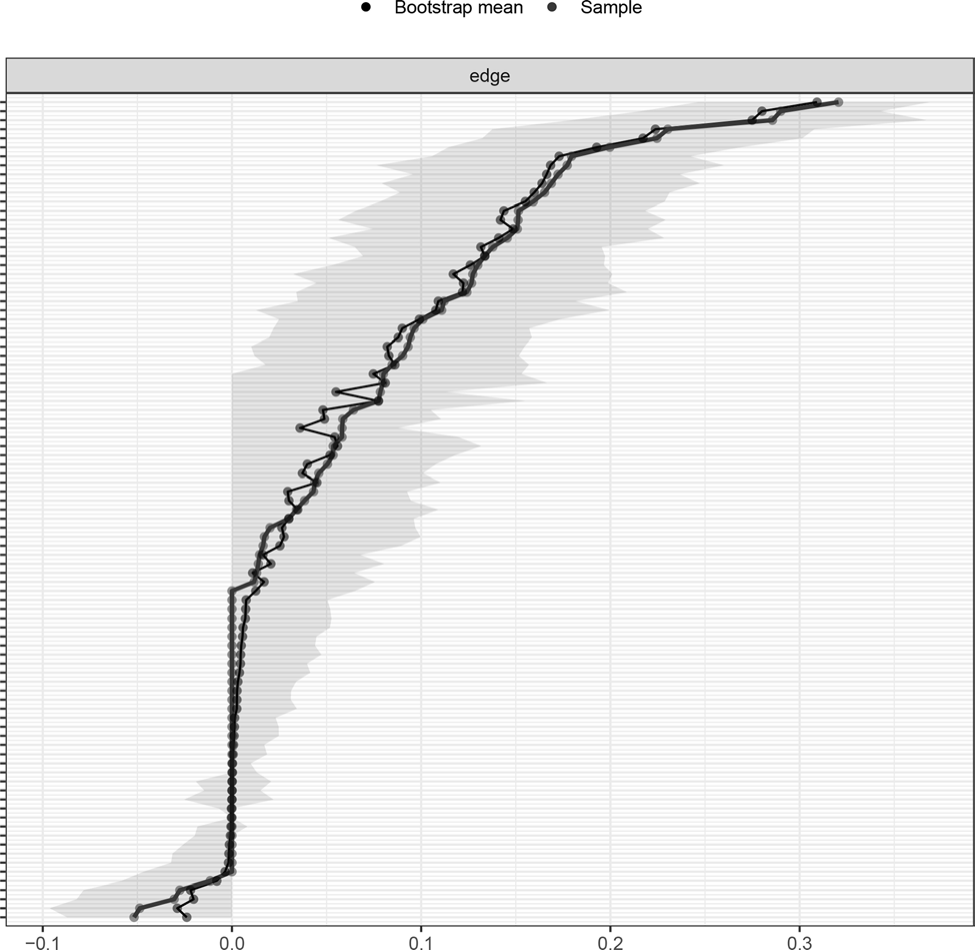
The 95% CIs of edge weights

As shown in Fig. 4, the CS coefficient for the bridge EI indices assessed by the case-dropping bootstrap method was 0.595 which indicated that the centrality indices remained stable after dropping 59.5% of the sample in the current study. More importantly, the relatively narrow 95% CIs for edge weights in Fig. 5 suggested that most edges were stable and accurate in the current network.

## Discussion

### Psychometric properties of the Chinese version of BWAS

Ground in the components model of addiction [5, 7], the 7-item BWAS [6] covers all core elements of addiction including salience, mood modification, tolerance, withdrawal, conflict, and relapse. In a sample of white-collar workers from a wide range of industries, we examined the psychometric properties of the Chinese version of BWAS. In line with studies using different language versions of BWAS in other non-Chinese samples [6, 11–15], the first aim of this study for testing the psychometric properties of BWAS among Chinese samples was achieved by revealing a one-factor structure with measurement invariance across genders, districts, and age groups, good reliability (e.g., internal consistency), and convergent and criterion validity. Our findings add to the literature that BWAS is a proper measurement tool for assessing WA in different cultural contexts including China.

In particular, the significantly positive correlations of BWAS with general anxiety and negative correlations with self-rated health status supported the scale’s criterion validity. In addition to occupation-related mental and physical health problems [40, 70, 71], our study shows the susceptibility of workaholics in China to general psychological and physical health problems. Prior studies have provided empirical evidence that workaholics frequently experience heightened general anxiety and poorer health status which have raised social concerns about employees’ well-being in both occupational and personal domains [16, 72]. Together with our study’s findings, organizations should be made aware that the long-term losses (e.g., poor job performance, more sickness absences, and increased job burnout as well as work-family conflict) associated with WA may cost more than its short-term benefits (e.g., long work hours, high work involvement and commitment, and high efficiency), resulting in less sustainable productivity [3, 73, 74]. Our findings have added to the empirical evidence regarding associated risk of WA and warranted organizations to further allocate resources to support interventions (e.g., cognitive behavioral therapy, rational emotive cognitive therapy, or meditation awareness training) [75] on mitigating WA for their long-term success.

### Work addiction and general anxiety

By network analysis, an advanced statistical approach to quantify the complex interactions between individual symptoms of the latent variables [37], the second aim of this study for identifying the comorbidity between WA and general anxiety was also achieved as we found that WA-tolerance and WA-problems were two central symptoms that play the most important role in the current network. Consistent with previous research [12] which found that the dominant element of WA is “tolerance”, the process by which the former effects are achieved by increasing amounts of the particular activity (e.g., working) [5, 7], our findings suggest that workaholics show a tendency to spend much more time on work than initially intended [2, 4, 6]. For instance, Griffiths (2011) [5] found a workaholic increased his work hours from 6 to 8 to 14 h per day in two years. The “problems” element of WA is defined as health harm due to overwork [4, 6] and occupied a central position in our network. This overwork-related health impairment has been commonly and persistently reported by prior studies [17, 70, 76] since the phenomenon “addiction to work” was initially introduced in academia [20]. Indeed, impaired functioning has been regarded as the most prominent diagnostic criteria for behavioral addictions in ICD-11 [77]. Organizational policies including setting flexible work schedules, restricting work hours per day (e.g., 8 h or less), promoting employees’ awareness of health problems associated with WA, as well as improving time management and preventive behaviors [3, 75, 78, 79] should be considered as part of the WA-mitigation program.

Results of our network analysis also demonstrated that WA-problems, WA-mood modification, and GA-mouth dryness were three bridge symptoms connecting different clusters of WA and general anxiety symptoms. It is not a surprise to find that WA-problems and mood modification, a coping strategy of overworking commonly used by workaholics to modify subjective negative experiences or affects (e.g., anxiety) [5–7], had the strongest correlations with GA-mouth dryness, a subjective feeling of autonomic physiological arousal that frequently accompanies general anxiety [80, 81]. These findings suggest that the relationship between WA and general anxiety may be maintained by the two critical pathways between their particular symptoms (i.e., problems-mouth dryness and mood modification-mouth dryness) in a vicious cycle. On one hand, workaholics’ health impairment (e.g., physical pain and psychological discomfort) brought by overwork will induce anxiety and activate their autonomic activities, including the feeling of mouth dryness [82, 83], which in turn may make them more worried about their health condition and exacerbate their overall health status [84–87]. On other hand, engaging in overworking has been adopted as an inflexible strategy for workaholics to cope with their negative affects including stress and worry, and such maladaptive work style also leads to various psychosocial and physical problems (e.g., burnout, work-family conflict, and physical pain [1, 17]) which further increase their overall anxiety level [88–90].

From the perspective of item-level network analysis [37], the potential interactions among individual symptoms of WA and general anxiety were used to depict a more comprehensive picture of both empirical evidences and intervention practices. To effectively lower the comorbidity between WA and general anxiety, both the three bridge symptoms (i.e., WA-problems, WA-mood modification, and GA-mouth dryness) and two bridge pathways (i.e., problems-mouth dryness and mood modification-mouth dryness) should be focused in intervention programs involving multiple strategies against WA and general anxiety. For examples, mouth dryness management (e.g., drinking water frequently and regularly and avoiding excessive caffeine and alcohol intake [80]) can be taught as a mean for symptom control while cognitive behavioral therapy may be incorporated for changing maladaptive core beliefs (e.g., overwork can modify negative feelings) [91].

### Implications

Our first aim to examine the psychometric properties of the Chinese version of BWAS provides statistical evidence to support the use of BWAS for assessing WA within the behavioral addiction framework [5, 7] in Chinese populations. This tool can be used in different industrial settings to identify not only potential risk/protective factors for WA but also a wide range of its adverse consequences among Chinese workers. Since BWAS has also now been validated in multiple languages including English, Chinese, Hungarian, Polish, Danish, Italian, and Turkish, cross-cultural comparisons are possible for better understanding of the socio-cultural differences on WA.

Furthermore, the two central symptoms and three bridge symptoms as well as two bridge pathways identified by network analysis (i.e., the second aim of this study) provide multiple intervention strategies for mitigating WA, general anxiety, as well as their comorbidity. For instance, preventive behaviors training [78] for WA-problems should be added to the existing intervention program for WA. Those bridge pathways connecting WA and anxiety are particularly helpful for alleviating WA and general anxiety simultaneously by combining intervention programs for both WA and general anxiety. More importantly, those programs should emphatically target at weakening the strong positive associations of mouth dryness with problems and mood modification.

### Limitations

The present study has several limitations that need to be further addressed. First, the cross-sectional design may be hard to infer the temporal directions of bridge pathways identified in the current WA-GA network and future research should conduct cross-lagged network analysis [92–94] to understand the symptom-to-symptom casualties underlying WA and general anxiety. Second, we collected responses from a specific worker subgroup (i.e., white-collar workers), and further studies may test the replicability of our findings in other employee groups (e.g., blue/red/grey/gold-collar workers) [95]. Third, recruiting participants through convenience sampling on the online data collection platform may have under-coverage bias by missing participants who did not register an account in this platform [96]. Thus, responses of a more representative sample including both online and offline participants should be considered in future research to avoid potential biased outcomes [96]. Fourth, a relatively small subsample of 50 participants was followed up and used to examine the test-retest reliability of BWAS, which warrants replication with a larger sample in future research [97]. Last but not least, this study examined how individual symptoms of WA and general anxiety interacted with each other. Further network analysis may be conducted to examine the comorbidity between WA and other common emotional problems (e.g., emotional exhaustion and depression) [1, 98] frequently reported by workaholics. Doing so may contribute to management practices for improving excessive working pattern and emotional wellbeing in the organizational context.

## Conclusions

The present findings support the use of BWAS in Chinese white-collar workers and demonstrated its satisfactory psychometric properties. Our validation of BWAS, which has multiple language versions, provides a useful tool for detecting WA of white-collar workers from various industries in the Chinese context, and may further inspire more WA-related studies in Chinese and other cultural settings. Furthermore, our network analysis results provided insights for the core symptoms for not only future empirical investigation but also cost-effective intervention of employees comorbid with WA and general anxiety. To conclude, the two aims of the current study were achieved, and these findings would help raise public, organizational, and employee awareness of WA-related consequences and improve employees’ well-being and health.

## Data Availability

The dataset used in the current study are available from the corresponding author on reasonable request.
